# Implementing organized colorectal cancer screening programs in Europe—protocol for a systematic review of determinants and strategies

**DOI:** 10.1186/s13643-023-02193-6

**Published:** 2023-02-27

**Authors:** Bianca Albers, Reto Auer, Laura Caci, Emanuela Nyantakyi, Ekaterina Plys, Clara Podmore, Franziska Riegel, Kevin Selby, Joel Walder, Lauren Clack

**Affiliations:** 1 Institute for Implementation Science in Health Care (IfIS), Medical Faculty, University of Zurich, Universitätstrasse 84, 8006 Zurich, Switzerland; 2 Institute of Primary Health Care (BIHAM), University of Bern, Mittelstrasse 43, 3012 Bern, Switzerland; 3 Center for primary care and public health (Unisanté), University of Lausanne, Rue de Bugnon 44, 1010 Lausanne, Switzerland; 4 Department of Infectious Diseases and Hospital Epidemiology, University Hospital Zurich, Rämistrasse 100, 8091 Zurich, Switzerland

**Keywords:** Systematic review, Cancer screening, Colorectal cancer, Bowel cancer, Implementation science, Implementation determinants, Implementation strategies

## Abstract

**Background:**

With a high mortality of 12.6% of all cancer cases, colorectal cancer (CRC) accounts for substantial burden of disease in Europe. In the past decade, more and more countries have introduced organized colorectal cancer screening programs, making systematic screening available to entire segments of a population, typically based on routine stool tests and/or colonoscopy. While the effectiveness of organized screening in reducing CRC incidence and mortality has been confirmed, studies continuously report persistent program implementation challenges. This systematic review will synthesize the literature on organized CRC screening programs. Its aim is to understand what is currently known about the barriers and facilitators that influence the implementation of these programs and about the implementation strategies used to navigate these determinants.

**Methods:**

A systematic review of primary studies of any research design will be conducted. CENTRAL, CINAHL, EMBASE, International Clinical Trials Registry Platform, MEDLINE, PsycINFO, and Scopus will be searched. Websites of (non-)government health care organizations and websites of organizations affiliated with authors of included studies will be screened for unpublished evaluation reports. Existing organized CRC screening programs will be contacted with a request to share program-specific grey literature. Two researchers will independently screen each publication in two rounds for eligibility. Included studies will focus on adult populations involved in the implementation of organized CRC screening programs and contain information about implementation determinants/ strategies. Publications will be assessed for their risk of bias. Data extraction will include study aim, design, location, setting, sample, methods, and measures; program characteristics; implementation stage, framework, determinants, strategies, and outcomes; and service and other outcome information. Findings will be synthesized narratively using the three stages of thematic synthesis.

**Discussion:**

With its sole focus on the *implementation* of *organized* CRC screening programs, this review will help to fill a central knowledge gap in the literature on colorectal cancer screening. Its findings can inform the decision-making in policy and practice needed to prioritize resources for establishing new and maintaining existing programs in the future.

**Systematic review registration:**

PROSPERO (CRD42022306580).

**Supplementary Information:**

The online version contains supplementary material available at 10.1186/s13643-023-02193-6.

## Background

With more than 500,000 yearly cases and a mortality of 12.6% of all cancer cases, colorectal cancer (CRC) accounts for a substantial burden of disease in Europe [1, 2]. A recent study estimated the costs of this burden in Europe, attributed to loss of productivity and the provision of different health care services, to have been €19.1 billion in 2015 [3]. Lifestyle related factors such as an unhealthy diet and sedentary behavior increase the risk of developing colorectal cancer, which typically begins with adenoma detectable by colonoscopy and progresses to invasive cancer over the course of 10 to 15 years [4, 5]. Based on these characteristics, it is not surprising that the pro-active, systematic, and early detection of colorectal cancer—rather than its re-active treatment—has become a priority on health policy agendas in and across European countries. In February 2021, the European Commission presented *Europe's Beating Cancer Plan*, including the goal to develop a new *EU Cancer Screening Scheme* with the aim to ensure that 90% of the eligible target population in Europe is offered a colorectal cancer screening by 2025 [6]. An additional driver of this push for enhancing preventive colorectal cancer services in Europe has been the realization that the target uptake rate of 65% among those eligible for a CRC screening—typically those aged 50 to 74—has not been reached [7]. This was the case despite remarkable progress in increasing the number of organized CRC screening programs across Europe, which are now available in 20 out of 27 countries in the European Union (EU) [6].

*Organized* screening programs offer systematic screening, typically based on different types of routine stool tests and/or colonoscopy, to entire segments of the population of a country or a region. Where organized screening programs do not exist, *opportunistic* screening is often used, i.e., screening that is not systematically monitored and which depends on primary care physician recommendations or patient requests [8]. Further characteristics of organized programs are: explicit policies that outline program offerings; a clearly defined target population; and the specification of guidelines for program administration, including instructions for implementing, monitoring, and assuring program quality, and the follow up of patients with positive screening results [5]. While the effectiveness of organized screening in reducing CRC incidence [2] and mortality [9–11] has been confirmed, studies continuously report persistent implementation challenges preventing organized CRC screening from reaching its full preventative potential. These include patient-related barriers to participating in screening [12–14]; provider-related barriers to promoting and engaging in screening [15, 16]; and system-related barriers to establishing and maintaining screening programs [15–17]. As a consequence of these challenges, public health authorities and health care professionals implementing cancer screening programs may not achieve the goals that they have set for the use of this otherwise research-supported intervention. This is especially critical for organized, i.e., publicly funded screening programs. The legitimacy of these programs in the public eye will depend on their demonstrated efficiency and effectiveness, and in the absence hereof funding may be discontinued, and evidence-based practice diminished [18, 19].

Therefore, there is an urgent need to better understand current best knowledge on the implementation of organized CRC screening programs. Few scholars have aimed to synthesize this knowledge base for *organized* screening programs only [14, 16, 20–22], and of these, none have solely focused on the entire range of implementation conditions for colorectal cancer screening programs—despite important differences in these conditions existing for organized screening programs in general and for organized *colorectal* cancer screening programs in particular. Organized screening programs in general require the use of population registers and monitoring systems, they depend on substantial negotiation with and joint engagement of multiple groups of health care professionals, and on the systematic development of capacity to maintain program activities over time. The multiple screening modalities available as part of many organized CRC screening programs add an additional layer of implementation complexity that differentiates this type of cancer screening from others. While programs may aim at reducing this complexity by only offering one screening modality, opportunistic screening based on alternative modalities continues to occur outside of programs [15, 23]. Furthermore, CRC screening modalities are often viewed as unattractive by eligible program participants making it particularly challenging to build and maintain program reach [14, 24].

In the field of implementation science, models and methods have been developed to systematically examine implementation in health care. Among these, implementation *determinants* and *strategies* are central concepts. Implementation determinants are the barriers and facilitators that, at different levels of an implementing system, can influence the outcomes of an implementation [25, 26]. To address and/or navigate these, health care professionals and their organizations depend on purposely crafted implementation strategies, i.e., methods and activities used to enhance the uptake, implementation and sustainment of evidenced interventions and services [27]. They are the means with which program robustness can be established, and obstacles and challenges to CRC screening program implementation be overcome [21, 28]. Ideally, implementation strategies are selected and designed prospectively, based on both a detailed analysis of anticipated implementation determinants and on knowledge about which strategies are best suited to target these determinants. This knowledge is still lacking and has led to calls for enhancing implementation strategy research in health and human services [29, 30]. In the field of CRC screening, this research has grown in recent years, with an increasing number of studies examining the use of implementation strategies [31–33], their feasibility [34, 35] or ability to improve program outcomes [36, 37]. However, due to a lack of synthesis, it is unclear to what degree this slowly accumulating knowledge base is related to organized CRC screening programs, and how it can contribute to improving their implementation.

Therefore, the aim of this review is to synthesize the literature on implementing organized colorectal cancer screening programs developed for adult populations at average risk for colorectal cancer and to examine, what is currently known about

- The barriers and facilitators, i.e., determinants, that influence the implementation of these programs in Europe, and about
- The implementation strategies used to navigate these determinants.

## Methods/design

This systematic review has been registered with the International Prospective Register of Systematic Reviews (PROSPERO, https://www.crd.york.ac.uk/prospero/, registration number: CRD42022306580). The review protocol is being reported using the Preferred Reporting Items for Systematic Reviews and Meta-Analyses Protocol (PRISMA-P) checklist [38], which is available as Additional file 1.

The review is an element of the research project *Improving Organized Colorectal Cancer Screening: An Implementation science study* (OCCSI). Funded by *Swiss Cancer Research*, and based on a mixed-methods design, OCCSI aims to build an understanding of current practices in implementing organized CRC screening programs in Switzerland. Enforced by a decentralized political structure, Swiss organized CRC screening programs are established at a cantonal level. Since the development of the first Swiss organized CRC screening program in 2013, about half of all 26 Swiss cantons have followed^1^. The majority of these programs were established in the years since 2019 and are in early stages of their implementation. This review will contribute to building the knowledge base on how to optimize this implementation and that of further programs to be established in Switzerland in the future.

The review is designed as a systematic integrative review (SIR), building on the SIR framework by Whittemore and Knafl [39], allowing for the inclusion of quantitative as well as qualitative study designs.

### Criteria for considering studies for this review

Criteria for selecting studies for this review were developed based on the SPIDER tool [40] and include: sample, phenomenon of interest, design, evaluation, and research type.

#### Sample

Studies focused on adult populations who are involved in the implementation of European organized colorectal cancer screening programs developed for individuals at average risk for colorectal cancer will be included in this systematic review. Among relevant study populations are, for example, program participants/recipients, health care professionals such as general practitioners or gastroenterologists, program administrators/coordinators, leaders, policy developers, politicians, funders, or individuals in other roles. “Implementation” refers to any activities undertaken to establish, improve, or sustain an organized CRC screening program. European countries are those that are considered part of the European Region of the World Health Organization (WHO)^2^.

#### Phenomenon of interest

The characteristics of organized CRC screening programs developed by the International Agency for Research on Cancer [41] will be applied. These include for screening to be organized at a national or regional level, and to be based on an explicit policy. They also imply for a central team to be responsible for organizing the program, the systematic participant invitation based on prespecified target populations, and the health care services provided within the program. The existence of a structure for program quality assurance is a further characteristic.

#### Design

Published primary studies, i.e., studies reporting original, new data, of any research design will be eligible for this review.

#### Evaluation

Information about (a) determinants, i.e., factors perceived or empirically reported to influence the implementation of organized CRC screening programs and (b) the strategies used to navigate these determinants are the central findings of interest for this review. Implementation strategies will be defined as the methods or techniques used to enhance the adoption, implementation, or sustainment of organized CRC screening programs [27].

Determinants may influence program implementation in positive or negative direction and affect, e.g., program reach, engagement of health care professionals, program implementation speed, funding availability or security, and other implementation characteristics and outcomes. In identifying strategies, any activities aimed at navigating, removing, or utilizing these determinants will be included. Information about determinants and strategies may be reported as perceptions, characteristics, views, experiences, or in other either qualitative or quantitative formats.

#### Research type

This review will include qualitative, quantitative, and mixed methods peer-reviewed primary studies. Grey literature will be limited to evaluation reports issued by government and non-government organizations.

### Information sources and search strategy

The databases below will be searched for studies published from January 2000 onward. This cut-off year was chosen based on a review of the commencement years for organized CRC screening programs in Europe listed in the International Agency for Research on Cancer’s (IARC) Handbook on Colorectal Cancer Screening [41]. This review showed that all but one program had been established after 2000. The exception was Italy, where organized CRC screening began in Florence in 1982. All other regions commenced screening in 2000 or later, confirming that the central evidence base in focus of this review would be included in studies published in or after this year.

- Cochrane Central Register of Controlled Trials (CENTRAL)
- Cumulative Index to Nursing and Allied Health Literature (CINAHL)
- EMBASE
- International Clinical Trials Registry Platform (ICTRP)
- MEDLINE
- PsycINFO
- Scopus

Search strategies to be used are outlined in Additional file 2.

### Supplementary searches

Given the central position of (non-)government health care organizations in initiating and implementing organized colorectal cancer screening programs in Europe, websites of organizations known to be engaged in promoting and scaling cancer screening efforts will be screened for unpublished evaluation reports. The final list of websites will include but not be limited to:

- Association of European Cancer Leagues
- Cancer Research UK
- Digestive Cancers Europe (DICE)
- European Cancer Organization
- European Cancer Patient Coalition
- International Agency for Research on Cancer (IARC)
- Innovative Partnership for Action Against Cancer (iPAAC)
- National Institute for Health and Care Excellence (NICE)
- Medical Research Council (MRC)
- UK National Screening Committee (UK NCS)
- World Endoscopy Organization (WEO)

Websites of organizations affiliated with authors of included studies will also be searched for eligible publications. Furthermore, reference lists of previous systematic reviews identified through the search process together with reference lists of included studies will be screened for relevant literature. Finally, existing organized CRC screening programs [41] will be contacted with a request to share program-specific grey literature.

### Language of publication

Studies written in Danish, English, French, German, Italian, Norwegian, Spanish, or Swedish will be included.

### Data collection and management

Following the upload of all references to the online systematic review application Covidence^3^, all screening work will occur on this platform. A PRISMA flowchart will be developed to summarize the inputs and results of each stage of the screening process.

### Study selection

Two researchers will independently screen each publication in two rounds, one focused on titles and abstracts and one on full texts. Both rounds will involve the testing of a screening protocol, guiding the decision-making of individual researchers. Researchers will test each protocol independently on a sample of ten studies, compare results and discuss (a) the degree to which the protocol requires refinement, and (b) final decision-making on the respective study. Protocols will then be refined and prepared for use with the remaining studies. Individual researchers will apply eligibility criteria independently and be blind to each other’s decisions about the in- or exclusion of publications. Disagreements between their individual judgements will be resolved by consensus or a third researcher.

### Quality appraisal

All included publications will be assessed for their risk of bias using checklists developed by JBI^4^ for different study designs, including randomized controlled trials, qualitative, and economic evaluations.

### Data extraction

Pairs of researchers will independently extract data from 10% of the studies. Data extraction results will be compared, and disagreements discussed in our research team to achieve full consensus on how to approach data extraction among all research team members.

Data from the remaining 90% of the studies will be extracted independently by one researcher, and the extraction checked, and quality assured by the lead author. The following information will be extracted: study aim; study design; location; methods; study settings; study sample; CRC screening program characteristics; implementation stage; implementation framework use; study measures; implementation determinant information; implementation strategy information; implementation, service and other outcomes that are related to determinants and/or strategies; information needed to conduct risk of bias assessment (design dependent). Implementation strategy information extraction will be based on the “Prerequisites to Measuring Implementation Strategies” (PMIS) framework [27], recommending for implementation strategies to be specified by name, included components, actors, actions, action targets, temporality, dose, implementation outcome targets, and justification. To enable the coding process described below, both implementation determinant and strategy information will be extracted in the form of entire sentences and/or text sections included in original articles.

Should missing data be detected during data extraction, lead authors of papers will be contacted for unreported data and/or any other additional details of interest to the research team.

### Data analysis

Findings will be synthesized narratively using the three stages of thematic synthesis [42]: (1) Line by line coding; (2) the development of descriptive themes; and [3] the generation of analytical themes.

As part of the *line-by-line coding* of extracted information, information on the determinants to and strategies for implementing organized CRC screening programs will be coded using both deductive and inductive coding. The deductive coding of determinants will be guided by the Consolidated Framework for Implementation Research, CFIR [25, 43] and that of implementation strategies by the Expert Recommendations for Implementing Change (ERIC) compilation of implementation strategies [44]. The CFIR is a framework developed to build the knowledge base on quality implementation of complex interventions and contains five domains of factors potentially influencing implementation processes. These include the intervention, the individuals involved in an implementation, the inner and outer setting of the implementation, and the implementation process. The CFIR has been widely used by scholars to categorize and analyze determinants to colorectal cancer screening efforts, both as part of systematic reviews [32, 45, 46] and of primary studies [47–49]. For this review, a recently updated version of the CFIR will be used [43].

The ERIC compilation of implementation strategies [44] includes the description of 73 implementation strategies commonly acknowledged as relevant activities that, when used separately or in combination, can facilitate the integration of evidenced interventions into routine health care and prevention. While scholars have pointed to limitations in using the ERIC compilation as a coding tool [50, 51] due to a lack of granularity characterizing some of its strategies, it represents the most comprehensive and varied list of implementation strategies available for coding purposes. It has been applied in multiple health care focused systematic reviews [52–54]. The parallel use of the PMIS framework [27] will further ensure that implementation strategies are thoroughly categorized and analyzed. Furthermore, the coding of strategies and determinants will also be inductive, allowing researchers to register any information not aligned with framework-based coding categories under the category “other”. Finally, researchers will be asked to develop brief memos of coding challenges related to framework use as they emerge, such that these can be addressed during regular team meetings.

Based on these considerations, a coding protocol will be developed. The coding protocol will be piloted by all members of the research team on a sample of five different studies. Researchers will identify potential limitations and needs for improvement individually and discuss these in a review team meeting to develop concrete steps for adjusting the coding protocol. The revised protocol will then be used for the coding of all included studies, with researchers having access to ad hoc support from other review team members and weekly research team meetings serving as an additional forum for problem solving around coding. These meetings will also be used to discuss inductive codes to enable decisions on whether to add new codes, and how to handle previously coded manuscripts.

To *develop descriptive themes*, two researchers will independently review the textual information retrieved for each code and develop descriptive themes by, e.g., identifying commonalities and differences in the ways in which implementation determinants and the use of implementation strategies are described; and by identifying how, e.g., different determinants, different strategies, and determinants and strategies are interlinked with each other. Descriptive themes will be based on the semantic level of texts, i.e., closely related to the language used in text excerpts. A summary of descriptive themes will be discussed among all members of the research team in two rounds—once at a preliminary stage to retrieve early input, and a second time to refine a more consolidated list of descriptive themes prior to their finalization.

In *developing analytical themes*, the research team will move to the latent level of meaning included in and across studies and build shared understandings of, e.g.,

- Commonalities in determinants to the implementation of organized CRC screening programs and how they impact this implementation
- Characteristics of implementation strategies used in the implementation of organized CRC screening programs
- Commonalities and differences in strategies selected to address specific determinants
- Linkages between determinants only, strategies only and determinants and strategies
- Gaps in the current knowledge base for the implementation of organized CRC screening programs

Preliminary ideas for analytical themes will be mirrored in descriptive themes and discussed in the research team also considering review findings and the wider literature on organized CRC screening program implementation. Based on this discussion, two researchers will draft a first list of potential analytical themes for further input from and discussion among all review team members. This will lead to further refinement and the finalization of all analytical themes.

### Involvement of program implementers

Organized Swiss colorectal cancer screening programs are at the center of the overarching OCCSI study, in which this review is embedded. Through two open, one-hour online meetings, individuals actively involved in implementing these programs will therefore be invited to participate in discussions about descriptive and analytic themes. Meeting invites will be issued twice—once when a first list of descriptive themes exists, and once at the outset of developing analytic themes—and e-mailed directly to the research team’s network of CRC screening program stakeholders. These meetings will be aimed at helping the research team understand program implementers’ perspective on preliminary review findings, thereby ensuring that these can be interpreted with these review users in mind. At a later stage of the review process, stakeholders will also be asked for feedback and input on the development of strategies for the wider dissemination of review findings.

## Discussion

In a new guide document, WHO Europe characterizes CRC screening as one of the “best buys” that exist in cancer screening [55]. The organization also recommends countries to build their screening efforts on *organized* programs with a capacity to reach at least 70% of an eligible population. This emphasizes the relevance of filling a gap in the literature on colorectal cancer screening by synthesizing the knowledge about how this type of program has been implemented thus far. Such synthesis can help to inform the decision-making in policy and practice needed to prioritize resources for establishing new and maintaining existing programs in the future.

One strength of this review will be to solely focus on studies conducted in the context of *organized* European CRC screening programs, thereby filtering out knowledge that has been created in contexts of opportunistic screening and/or of research trials that may involve systematic screening efforts but at a much smaller scale than that of entire populations in a region, state, or nation. A further strength is that all review stages will be informed by an implementation science-based perspective, reflected in the use of clearly delineated concepts—implementation determinants and strategies—and frameworks supporting their analysis in the context of CRC screening. In combination with the inclusion of a broad range of knowledge types—qualitative as well as quantitative—this will allow for generating a much-needed knowledge base that currently is missing for the implementation of organized colorectal cancer screening programs.

It is also expected that the review team will encounter some limitations when conducting this review. First, a substantial number of existing organized CRC screening programs in Europe listed in the most recent version of the IARC Handbook on Colorectal Cancer Screening [41] have been established in the past 10–12 years, a period during which the field of implementation science also underwent considerable growth and development. This may limit the number of genuine implementation studies conducted of organized CRC screening programs—a limitation that we will address by consulting complementary grey literature existing in the public domain but not indexed in electronic databases. Second, with organized CRC screening programs being at the core of this review, significant heterogeneity could make synthesis difficult. Included studies will have been conducted in a broad range of countries, representing different policy contexts, welfare state regimes and health care systems, making it difficult to compare and synthesize findings. Furthermore, CRC screening programs presented may be at different stages of their implementation (with some being in, e.g., a pilot phase, and others reporting on multiple years of implementation experience) or include different program components (with some providing, e.g., different types of stool tests only, and others also including colonoscopies). In our data extraction, analysis, and synthesis, we will work to take these contextual differences into account and report on them as deemed relevant. Finally, the inclusion of a broad range of study designs in this review implies that—when presenting findings—small scale case studies are assigned the same weight as large-scale population research, potentially skewing results. To address this concern, we will provide detailed information about the evidence quality of studies, thereby allowing review users to put findings into perspective. Moreover, it is worth keeping in mind that this review pursues the explorative aim of understanding what is currently known about the implementation determinants and strategies related to organized CRC screening programs, making it relevant to consider the broadest possible range of evidence.

This breadth will allow for review findings to inform policy processes through the greatest possible variety in determinants and strategies that are relevant to consider when planning for program resources, design, and implementation. Simultaneously, review results will be usable in existing organized CRC program practice by identifying promising approaches to implementing and maintaining programs over time, providing decision-makers with information on which aspects of program practice may need particular effort, or ongoing adjustment and improvement. The findings from this review will therefore be disseminated in multiple forms, including scientific publication, conference presentations and targeted formats to be developed in collaboration with the stakeholders participating in the overarching study to which this review is linked.

Should protocol amendments be needed in the future, these will be registered in the PROSPERO repository and reported in the final review report.

## Supplementary Information

**Additional file 1.** PRISMA-P 2015 Checklist.

**Additional file 2.** Search Strategy.

## Data Availability

Not applicable.

## Abbreviations

CFIR: Consolidated Framework for Implementation Research
CRC: Colorectal Cancer
ERIC: Expert Recommendations for Implementing Change
EU: European Union
IARC: International Agency for Research on Cancer
JBI: Joanna Briggs Institute
PMIS: Prerequisites to Measuring Implementation Strategies
WHO: World Health Organization

## Footnotes

1 An overview is available at https://www.swisscancerscreening.ch/de/angebote-in-ihrem-kanton

2 As listed on the WHO website at https://www.who.int/countries

3 Covidence systematic review software, Veritas Health Innovation, Melbourne, Australia. Available at www.covidence.org

4 JBI’s repository of quality appraisal tools can be accessed here: https://jbi.global/critical-appraisal-tools
