# Supplementary material for: Implementing organized colorectal cancer screening programs in Europe—protocol for a systematic review of determinants and strategies
Source: Syst Rev. 2023 Feb 27;12:26. doi: 10.1186/s13643-023-02193-6 (PMC9969690; doi:10.1186/s13643-023-02193-6)
Supplement: Supplementary file 2 — Additional file 2. Search Strategy. [file 13643_2023_2193_MOESM2_ESM.docx]

| **CINAHL** | |
| --- | --- |
| # | Query |
| S1 | (MH "Colorectal Neoplasms+") OR (MH "Sigmoid Neoplasms") OR (MH "Rectal Neoplasms") OR (MH "Occult Blood") OR (MH "Colonoscopy+") OR (MH "Sigmoidoscopy") OR TI((colorectal OR rectum OR rectal OR colon* OR sigmoid) N3 (cancer* OR malignancy OR neoplasm* OR tumor OR tumour OR carcinoma* OR adenocarcinoma*)) OR TI((fec* OR faec * OR stool OR "occult blood") N3 (analysis OR test OR examination)) OR TI(colonscop* OR coloscopy* OR sigmoidoscop* OR proctosigmoidoscopy* OR rectosigmoidoscop* OR sigmoideoscop* OR enteroscop*)OR TI((intestine* OR colon* OR rect*) N3 endoscop*) OR AB((colorectal OR rectum OR rectal OR colon* OR sigmoid) N3 (cancer* OR malignancy OR neoplasm* OR tumor OR tumour OR carcinoma* OR adenocarcinoma*)) OR AB((fec* OR faec * OR stool OR "occult blood") N3 (analysis OR test OR examination)) OR AB(colonscop* OR coloscopy* OR sigmoidoscop* OR proctosigmoidoscopy* OR rectosigmoidoscop* OR sigmoideoscop* OR enteroscop*) OR AB((intestine* OR colon* OR rect*) N3 endoscop*) |
| S2 | (((MH "Early Detection of Cancer") OR (MH "Cancer  Screening") OR TI((mass OR cancer OR crc) N1 screening) OR AB((mass OR cancer OR crc) N1 screening) OR TI(("early detection" OR "early diagnosis" OR "early cancer diagnosis" OR screening) N3 program*) OR AB(("early detection" OR "early diagnosis" OR "early cancer diagnosis" OR screening) N3 program*)) |
| S3 | TI(barrier* OR facilitator* OR obstacle* OR hurdle* OR  challenge* OR determinant* OR enabler*) OR TI((determ* OR imped* OR hamper* OR interfer* OR disturb* OR improv* OR increas* OR enhanc* OR promot* OR support* OR encourag* OR influenc*) N3 (adopt* OR participat* OR uptake OR implement*) N6 (program* OR screening)) OR AB(barrier* OR facilitator* OR obstacle* OR hurdle* OR challenge* OR determinant* OR enabler*) OR AB((determ* OR imped* OR hamper* OR interfer* OR disturb* OR improv* OR increas* OR enhanc* OR promot* OR support* OR encourag* OR influenc*) N3 (adopt* OR participat* OR uptake OR implement*) N6 (program* OR screening)) |
| S4 | S1 AND S2 AND S3 [*Limiters - Published Date: 20000101-; Language: Danish, English, French, German, Italian, Norwegian, Spanish, Swedish Expanders - Apply equivalent subjects; Search modes - Find all my search terms*] |

| **Embase** | |
| --- | --- |
| # | Query |
| 1 | 'colorectal cancer'/exp OR 'sigmoid cancer'/exp OR 'rectum cancer'/exp OR 'feces analysis'/exp OR 'occult blood test'/exp OR 'colonoscopy'/exp OR 'sigmoidoscopy'/exp OR 'rectoscopy'/exp OR (((colorectal OR rectum OR rectal OR colon* OR sigmoid) NEAR/3 (cancer* OR malignancy OR neoplasm* OR tumor OR tumour OR carcinoma* OR adenocarcinoma*)):ti,ab) OR (((fec* OR faec * OR stool OR 'occult blood') NEAR/3 (analysis OR test OR examination)):ti,ab) OR colonscop*:ti,ab OR coloscopy*:ti,ab OR sigmoidoscop*:ti,ab OR proctosigmoidoscopy*:ti,ab OR rectosigmoidoscop*:ti,ab OR sigmoideoscop*:ti,ab OR enteroscop*:ti,ab OR (((intestine* OR colon* OR rect*) NEAR/3 endoscop*):ti,ab) |
| 2 | 'cancer screening'/exp/mj OR 'mass screening'/exp/mj OR (((((mass OR cancer OR crc) NEAR/1 screening):ti,ab) OR ((('early detection' OR 'early diagnosis' OR 'early cancer diagnosis' OR screening) NEAR/3 program*):ti,ab)) AND ('organization and management'/exp OR 'public health'/exp OR 'health program'/exp OR outreach:ti,ab OR population:ti,ab OR organized:ti,ab OR organised:ti,ab OR federal*:ti,ab)) |
| 3 | barrier*:ti,ab,kw OR facilitator*:ti,ab,kw OR obstacle*:ti,ab,kw OR hurdle*:ti,ab,kw OR challenge*:ti,ab,kw OR determinant*:ti,ab,kw OR enabler*:ti,ab,kw OR (((determ* OR imped* OR hamper* OR interfer* OR disturb* OR improv* OR increas* OR enhanc* OR promot* OR support* OR encourag* OR influenc*) NEAR/3 (adopt* OR participat* OR uptake OR implement*) NEAR/6 (program* OR screening)):ti,ab,kw) |
| 4 | #1 AND #2 AND #3 |
| 5 | #1 AND #2 AND #3 AND [conference abstract]/lim |
| 6 | #1 AND #2 AND #3 NOT [conference abstract]/lim |
| 7 | #1 AND #2 AND #3 NOT [conference abstract]/lim AND [2000-2022]/py |
| 8 | #1 AND #2 AND #3 NOT [conference abstract]/lim AND [2000-2022]/py AND ([danish]/lim OR [english]/lim OR [french]/lim OR [german]/lim OR [italian]/lim OR [norwegian]/lim OR [spanish]/lim OR [swedish]/lim) |

| **Medline** | |
| --- | --- |
| # | Query |
| S1 | (MH "Colorectal Neoplasms+") OR (MH  "Sigmoid Neoplasms") OR (MH "Rectal Neoplasms+") OR (MH "Occult Blood") OR (MH "Colonoscopy+") OR (MH "Sigmoidoscopy") OR TI((colorectal OR rectum OR rectal OR colon* OR sigmoid) N3 (cancer* OR malignancy OR neoplasm* OR tumor OR tumour OR carcinoma* OR adenocarcinoma*)) OR TI((fec* OR faec * OR stool OR "occult blood") N3 (analysis OR test OR examination)) OR TI(colonscop* OR coloscopy* OR sigmoidoscop* OR proctosigmoidoscopy* OR rectosigmoidoscop* OR sigmoideoscop* OR enteroscop*)OR TI((intestine* OR colon* OR rect*) N3 endoscop*) OR AB((colorectal OR rectum OR rectal OR colon* OR sigmoid) N3 (cancer* OR malignancy OR neoplasm* OR tumor OR tumour OR carcinoma* OR adenocarcinoma*)) OR AB((fec* OR faec * OR stool OR "occult blood") N3 (analysis OR test OR examination)) OR AB(colonscop* OR coloscopy* OR sigmoidoscop* OR proctosigmoidoscopy* OR rectosigmoidoscop* OR sigmoideoscop* OR enteroscop*) OR AB((intestine* OR colon* OR rect*) N3 endoscop*) |
| S2 | (MH "Early Detection of Cancer") OR (MH "Mass Screening+") OR ((TI((mass OR cancer OR crc) N1 screening) OR AB((mass OR cancer OR crc) N1 screening) OR TI(("early detection" OR "early diagnosis" OR "early cancer diagnosis" OR screening) N3 program*) OR AB(("early detection" OR "early diagnosis" OR "early cancer diagnosis" OR screening) N3 program*)) AND ((MH "Organization and Administration") OR (MH "Public Health") OR (MH "Public Health Administration") OR (MH "National Health Programs") OR (MH "Preventive Health Services") OR TI(outreach OR population OR organized OR organised OR federal*) OR AB(outreach OR population OR organized OR organised OR federal*))) |
| S3 | TI(barrier* OR facilitator* OR obstacle* OR hurdle* OR  challenge* OR determinant* OR enabler*) OR TI((determ* OR imped* OR hamper* OR interfer* OR disturb* OR improv* OR increas* OR enhanc* OR promot* OR support* OR encourag* OR influenc*) N3 (adopt* OR participat* OR uptake OR implement*) N6 (program* OR screening)) OR AB(barrier* OR facilitator* OR obstacle* OR hurdle* OR challenge* OR determinant* OR enabler*) OR AB((determ* OR imped* OR hamper* OR interfer* OR disturb* OR improv* OR increas* OR enhanc* OR promot* OR support* OR encourag* OR influenc*) N3 (adopt* OR participat* OR uptake OR implement*) N6 (program* OR screening)) |
| S4 | S1 AND S2 AND S3 |
| S5 | S1 AND S2 AND S3 [*Limiters - Date of Publication: 20000101-; Language: Danish, English, French, German, Italian, Norwegian, Spanish, Swedish; Expanders - Apply equivalent subjects; Search modes - Find all my search terms*] |

| **PsycINFO** | |
| --- | --- |
| # | Query |
| S1 | TI((colorectal OR rectum OR rectal OR colon* OR sigmoid) N3 (cancer* OR malignancy OR neoplasm* OR tumor OR tumour OR carcinoma* OR adenocarcinoma*)) OR TI((fec* OR faec * OR stool OR “occult blood”) N3 (analysis OR test OR examination)) OR TI(colonoscop* OR colonoscopy* OR sigmoidoscop* OR proctosigmoidoscopy* OR rectosigmoidoscop* Or sigmoideoscop* OR enteroscop*) OR TI((intestine* OR colon* OR rect*) N3 endoscop*) OR AB((colorectal OR rectum OR rectal OR colon* OR sigmoid) N3 (cancer* OR malignancy OR neoplasm* OR tumor OR tumour OR carcinoma* OR adenocarcinoma*)) OR AB((fec* OR faec * OR stool OR “occult blood”) N3 (analysis OR test OR examination)) OR AB(colonoscop* OR coloscopy OR sigmoidoscop* OR proctosigmoidoscopy* OR rectosigmoidoscop* Or sigmoideoscop* OR enteroscop*) OR AB((intestine* OR colon* OR rect*) N3 endoscop*) |
| S2 | (((DE “Cancer Screening”) OR TI((mass OR cancer OR crc) N1 screening) OR AB((mass OR cancer OR crc) N1 screening OR TI((“early detection” OR “early diagnosis” OR “early cancer diagnosis” OR screening) N3 program*) OR AB((((“early detection” OR “early diagnosis” OR “early cancer diagnosis” OR screening) N3 program*)) AND ((DE “public Health” OR DE “Public Health Campaigns”) OR TI(outreach OR population OR organized OR organized OR federal*) OR AB((outreach OR population OR organized OR organized OR federal*))) |
| S3 | (DE “Treatment Barriers“) OR (DE “Participation“) OR TI(barrier* OR obstacle* OR hurdle* OR challenge* OR determinant* OR enabler*) OR TI((determ* OR imped* OR hamper* OR interfer* OR disturb* OR improv* OR increase* OR enhance* OR promot* OR support* OR encourage* OR influenc*) N3 (adopt* OR participat* OR uptake OR implement*) N6 (program* OR screening)) OR AB(barrier* OR obstacle* OR hurdle* OR challenge* OR determinant* OR enabler*) OR AB((determ* OR imped* OR hamper* OR interfer* OR disturb* OR improv* OR increase* OR enhance* OR promot* OR support* OR encourage* OR influenc*) N3 (adopt* OR participat* OR uptake OR implement*) N6 (program* OR screening)) |
| S4 | S1 AND S2 AND S3 |
| S5 | S1 AND S2 AND S3 [*Limiters – Publication Year: 2000-; Language: Danish, English, French, German, Italian, Norwegian, Spanish, Swedish; Expanders – Apply equivalent subjects; Search modes – Find all my search terms*] |

| **Scopus** | |
| --- | --- |
| # | Query |
| S1 | (TITLE-ABS-KEY ( ( colorectal OR rectum OR rectal OR colon* OR sigmoid ) W/3  (cancer* OR malignancy OR neoplasm* OR tumor OR tumour OR carcinoma* OR adenocarcinoma* ) ) OR TITLE-ABS-KEY ( ( fec* OR faec * OR stool OR "occult blood" ) W/3 ( analysis OR test OR examination ) ) OR TITLE-ABS-KEY ( colonscop* OR coloscopy* OR sigmoidoscop* OR proctosigmoidoscopy* OR rectosigmoidoscop* OR sigmoideoscop* OR enteroscop* ) OR TITLE-ABS-KEY ( ( intestine* OR colon* OR rect* ) W/3 endoscop* ) ) AND ( ( TITLE-ABS-KEY ( ( mass OR cancer OR crc ) W/1 screening ) OR TITLE-ABS-KEY ( ( "early detection" OR "early diagnosis" OR "early cancer diagnosis" OR screening ) W/3 program* ) ) AND TITLE-ABS-KEY ( outreach OR population OR organized OR organized OR federal* ) ) AND ( TITLE-ABS-KEY ( barrier* OR facilitator* OR obstacle* OR hurdle* OR challenge* OR determinant* OR enabler* ) OR TITLE- ABS-KEY ( ( determ* OR imped* OR hamper* OR interfer* OR disturb* OR improv* OR increas* OR enhanc* OR promot* OR support* OR encourag* OR influenc* ) W/3 ( adopt* OR participat* OR uptake OR implement* ) W/6 ( program* OR screening ) ) ) AND ( LIMIT-TO ( PUBYEAR , 2022 ) OR LIMIT-  TO ( PUBYEAR , 2021 ) OR LIMIT-TO ( PUBYEAR , 2020 ) OR LIMIT-TO ( PUBYEAR , 2019 ) OR LIMIT-TO ( PUBYEAR , 2018 ) OR LIMIT-TO ( PUBYEAR , 2017 ) OR LIMIT- TO ( PUBYEAR , 2016 ) OR LIMIT-TO ( PUBYEAR , 2015 ) OR LIMIT-TO ( PUBYEAR , 2014 ) OR LIMIT-TO ( PUBYEAR , 2013 ) OR LIMIT-TO ( PUBYEAR , 2012 ) OR LIMIT- TO ( PUBYEAR , 2011 ) OR LIMIT-TO ( PUBYEAR , 2010 ) OR LIMIT-TO ( PUBYEAR , 2009 ) OR LIMIT-TO ( PUBYEAR , 2008 ) OR LIMIT-TO ( PUBYEAR , 2007 ) OR LIMIT- TO ( PUBYEAR , 2006 ) OR LIMIT-TO ( PUBYEAR , 2005 ) OR LIMIT-TO ( PUBYEAR , 2004 ) OR LIMIT-TO ( PUBYEAR , 2003 ) OR LIMIT-TO ( PUBYEAR , 2002 ) OR LIMIT- TO ( PUBYEAR , 2001 ) OR LIMIT-TO ( PUBYEAR , 2000 ) ) AND ( LIMIT-TO ( LANGUAGE , "English" ) OR LIMIT-TO ( LANGUAGE , "French" ) OR LIMIT-TO ( LANGUAGE , "Spanish" ) OR LIMIT-TO ( LANGUAGE , "German" ) OR LIMIT-  TO ( LANGUAGE , "Italian" ) ) |
